# Wildtype peers rescue social play and 50-kHz ultrasonic vocalization deficits in juvenile female *Cacna1c* heterozygous rats

**DOI:** 10.3389/fnbeh.2023.1190272

**Published:** 2023-08-03

**Authors:** Rebecca Bogdan, Rukhshona Kayumova, Rainer K. W. Schwarting, Markus Wöhr, Theresa M. Kisko

**Affiliations:** ^1^Behavioral Neuroscience, Experimental and Biological Psychology, Faculty of Psychology, Philipps-University Marburg, Marburg, Germany; ^2^Centre for Mind, Brain, and Behavior (CMBB), Philipps-University Marburg, Marburg, Germany; ^3^Social and Affective Neuroscience Research Group, Laboratory of Biological Psychology, Research Unit Brain and Cognition, Faculty of Psychology and Educational Sciences, KU Leuven, Leuven, Belgium; ^4^Leuven Brain Institute, KU Leuven, Leuven, Belgium

**Keywords:** Ca_v_1.2, calcium, rough-and-tumble play, ultrasonic communication, social environment, development

## Abstract

**Background:**

Healthy brain development depends on early social practices and experiences. The risk gene *CACNA1C* is implicated in numerous neuropsychiatric disorders, in which key characteristics include deficits in social functioning and communication. Recently, we reported sex-dependent impairments in social behavior and ultrasonic vocalizations (USV) in juvenile heterozygous *Cacna1c*^+/−^ (HET) rats. Specifically, HET females displayed increases in rough-and-tumble play that eliminated the typically observed sex difference between male and female rats. Interestingly, female wild-type *Cacna1c*^+/+^ (WT) pairs also showed a similar increase in social play when housed with HET females, suggesting their behavior may be influenced by HET cage mates. This indicates that the genetic makeup of the social environment related to *Cacna1c* can influence social play, yet systematic studies are lacking.

**Methods:**

In the present study, we housed juvenile females in MIXED- or SAME-genotype cages and tested them in a social play paradigm with a same- and opposite-genotype partner.

**Results:**

The results show that the early social environment and the genotype of the play partner influence social play and 50-kHz USV emission. Experience with a WT play partner appears necessary for HET females to show comparable levels of play and 50-kHz USV emission. Same-genotype HET pairs played less and emitted fewer 50-kHz USV than same-genotype WT or opposite-genotype pairs; however, we found that the decrease in social play and 50-kHz USV in HET pairs can be rescued by playing with a WT partner. The effect was particularly prominent when the first play partner was WT, as we found it increased play and 50-kHz USV emission in all subsequent interactions with ensuing partners.

**Conclusion:**

These findings suggest that the genetic makeup related to the social environment and/or social peers influences social play in *Cacna1c*^+/−^ haploinsufficient rats. Specifically, our results show that WT peers can rescue behavior and communication alterations in *Cacna1c* female rats. Our findings have important implications because they show that the genetic makeup of the social environment can divulge phenotypic changes in genetic rat models of neuropsychiatric disorders.

## 1. Introduction

In humans, the risk gene *CACNA1C* has been linked through genome-wide association studies (GWAS) to neuropsychiatric disorders with prominent social deficits, such as autism spectrum disorder (ASD) (Green et al., 2010; Cross-Disorder Group of the Psychiatric Genomics Consortium, 2013). The *CACNA1C* gene encodes the Ca_v_1.2 L-type calcium channel and is an auspicious therapeutic target (Zamponi, 2016). In humans, the single-nucleotide polymorphism rs1006737 has been correlated to atypical social behavior and communication (Krug et al., 2010; Dima et al., 2013; Pasparakis et al., 2015). In genetic *Cacna1c* rodent models, social deficits in behavior and communication have been found in mice (Bader et al., 2011; Kabir et al., 2016) and rats (Kisko et al., 2018, 2020; Redecker et al., 2019; Wöhr et al., 2020).

Due to rats’ gregarious nature, they are a widely used model species for studying social behavior and communication (Homberg et al., 2017). As juveniles, rats interact with peers through rough-and-tumble play, also called social play (Panksepp et al., 1984; Pellis and Pellis, 2009). Rough-and-tumble play is essential for proper brain and behavioral development in rats (Pellis and Pellis, 2009; Vanderschuren and Trezza, 2013; Vanderschuren et al., 2016). Comprised of complex, fast-paced back-and-forth competition for access to each other’s nape using components such as pinning, wrestling, and chasing, social play is an intricate combination and coordination of behavior and communication that requires complex but finely timed interactions of numerous neural systems (Vanderschuren et al., 2016).

Acoustic communication through ultrasonic vocalizations (USV) is another essential factor in the social repertoire of rats. Juvenile and adult rats emit two main types of USV: aversive 22-kHz and appetitive 50-kHz calls. Aversive 22-kHz calls are associated with negative affective states and occur mainly in aversive contexts, such as exposure to predators or pain, but also during defensive and submissive displays in intermale fighting (Blanchard et al., 1991; Browning et al., 2017). On the other hand, 50-kHz USV reflect a positive affective state and is also known as “rat laughter” (Panksepp, 2005). Positive 50-kHz calls occur in appetitive and rewarding situations (Burgdorf et al., 2008; Brudzynski, 2013), most notably during rough-and-tumble play (Knutson et al., 1998). Moreover, systemic administration of psychoactive substances, such as amphetamine, can elicit 50-kHz USV at very high rates indicative of a mania-like state (Wöhr, 2022). During social play in rats, 50-kHz USV are important for facilitating and maintaining a playful mood (Himmler B. et al., 2014; Kisko et al., 2015b). Rats specifically bred for low emissions of 50-kHz USV show reduced social play behavior (Burgdorf et al., 2013), and devocalization studies show that in rats unable to emit USV, social play behavior is severely diminished (Kisko et al., 2015b).

Social behavior and USV in combination convey considerable information on the animal’s affective state and social motivation and how they respond to various social peers. Therefore, using social behavior in conjunction with USV seems to be a particularly suitable tool for studying genetic and environmental risk factors in rodent models for neuropsychiatric disorders, such as ASD. Regarding genetics, studies have shown that different rat strains display differences in play (Himmler B. et al., 2014; Lukas and Wöhr, 2015; Northcutt and Nwankwo, 2018). Moreover, breeding for different anxiety levels in rats can result in differences in play behavior (Himmler B. et al., 2014; Lukas and Wöhr, 2015; Northcutt and Nwankwo, 2018). Relating to the effects of the environment, studies show that in rats the availability and characteristics of play partners has an important role (Poole and Fish, 1975; Hol et al., 1999; van den Berg et al., 1999; Pellis et al., 2018; Stark and Pellis, 2020). For example, control rats living with devocalized cage mates play 50% less than control rats living with non-devocalized cage mates (Kisko et al., 2015b). Additionally, as adults, juvenile rats that were reared with play-deprived partners have deficits in social functions (Hol et al., 1999; van den Berg et al., 1999). These examples provide compelling evidence that the makeup of cage mates during the critical play period can influence social play, social communication and socio-cognitive skills needed in adult social interactions (Bell et al., 2010; Baarendse et al., 2013; Himmler S. et al., 2014; Kisko et al., 2015a; Schneider et al., 2016; Pellis et al., 2017).

Our heterozygous *Cacna1c* model exhibits several characteristics reminiscent of ASD-like social and communication deficits in juveniles and adults (Schwarting et al., 2018; Redecker et al., 2019; Kisko et al., 2020). We recently found a sex-dependent influence of *Cacna1c* haploinsufficiency on social behavior and emission of 50-kHz USV in juvenile rats (Kisko et al., 2020, 2021). While social play behavior was not affected in males, 50-kHz USV emission during social play was reduced in *Cacna1c* heterozygous (HET) rats compared to *Cacna1c* wildtype (WT) controls. In contrast, female HET pairs played much more than WT controls, but 50-kHz USV emission was comparable. Sex-dependent effects are not altogether surprising as there has been ample evidence for sex-specific effects of the risk gene *CACNA1C* in humans (Dao et al., 2010; Strohmaier et al., 2013; Heilbronner et al., 2015) and *Cacna1c* mouse models (Dao et al., 2010). Most strikingly, however, was a lack of general sex difference in play behavior between male and female WT rats (Kisko et al., 2020). Since all animals investigated were housed in cages with both HET and WT cage mates and exclusively played with same-genotype partners, effects due to the peers within their social environment are conceivable. Thus, the social behavior of WT females may be influenced by the presence of hyper-playful HET cage mates.

Studies in mice have shown that elements of the home cage social environment can interact with genotype to impact particular aspects of disorder-related behaviors in ASD mouse models (Yang et al., 2015). For example, social deficits in an ASD mouse model can be rescued by rearing them with a highly social mouse strain (Yang et al., 2011). Consequently, using our *Cacna1c* haploinsufficient rat model, we controlled the cage mate’s genotype and the play partner’s genotype in juvenile females. We then investigated the interplay between acute and long-term effects of the genetic makeup related to the social environment and social peers on rough-and-tumble play and concomitant emission of 50-kHz USV in *Cacna1c* haploinsufficient rats, as compared to wildtype littermate controls. Implications from our findings highlight the importance of the early environment as a factor modulating phenotypic changes in genetic rat models for neuropsychiatric disorders.

## 2. Materials and methods

### 2.1. Animals and genotyping

The interplay between acute and long-term effects of the genetic makeup related to the social environment and social peers was assessed using rough-and-tumble play and concomitant emission of 50-kHz USV in a group of juvenile female *Cacna1c*^+/–^ (HET) rats (*N* = 40) and *Cacna1c*^+/+^ (WT) littermate controls (*N* = 40) by systematically manipulating the genotype of cage mates and the genotype of play partners, respectively.

As described in preceding studies (Kisko et al., 2018, 2020), HET rats were generated through zinc finger technology (Geurts et al., 2009) by SAGE Labs (now Envigo, Indianapolis, IN, USA) on a Sprague-Dawley (SD) background. HET rats carry a 4 base pair deletion at position 460 649-460 652 in genomic sequence, resulting in an early stop codon in exon 6. Genotyping was performed using DNA obtained from tail samples on a 3130xl Genetic Analyzer (Thermo Fisher Scientific, Waltham, MA, USA) with the following primers:

50-GCTGCTGAGCCTTTTATTGG-30 (*Cacna1c* Cel-1 F).

50-CCTCCTGGATAGCTGCTGAC-30 (*Cacna1c* Cel-1 R).

An established breeding protocol was applied to obtain HET and WT offspring, pairing SD females (Charles River, Sulzfeld, Germany) and male HET rats (Kisko et al., 2018). The day of birth was defined as postnatal day (PND) 0. Breeding was performed at the Faculty of Psychology, Philipps-University of Marburg, Germany.

Rats were identified by paw tattoo, using non-toxic animal tattoo ink (Ketchum permanent tattoo inks green paste, Ketchum Manufacturing Inc., Brockville, Canada). The ink was inserted subcutaneously through a 30-gauge hypodermic needle tip into the center of the paw on PND 3 ± 1. All procedures were conducted in strict accordance with the National Institutes of Health Guidelines for the Care and Use of Laboratory Animals and the relevant local or national rules and regulations of Germany and were subject to prior authorization by the local government (Tierschutzbehörde, Regierungspräsidium Gießen, Germany).

### 2.2. Experimental design

For our design we manipulated the genotype of the cage mate and the genotype of the play partner to fully assess the interplay between acute and long-term effects of the genetic makeup related to the social environment and social peers on rough-and-tumble play behavior and concomitant 50-kHz USV emission in juvenile *Cacna1c* haploinsufficient females.

#### 2.2.1. Genotype of the cage mate (SAME- genotype vs. MIXED-genotype cage)

On the day of weaning (PND 21), juvenile females were separated into experimental housing conditions in which the genotype of the cage mate was manipulated. Experimental housing consisted of social housing in groups of 4–6 with same-sex littermates in either SAME-genotype cages (all WT females or all HET females) or MIXED-genotype cages (equal distribution of WT and HET females). All females were housed in polycarbonate Macrolon Type IV cages (Tecniplast Deutschland GmbH, Hohenpeißenberg, Germany; 58 cm × 38 cm × 20 cm, length × width × height) and kept under standard laboratory conditions (22 ± 2°C and 40–70% humidity) with free access to standard rodent chow and water.

#### 2.2.2. Genotype of the play partner (WT vs. HET)

Forty WT and HET females from SAME- and MIXED-genotype cages were selected as focus rats and were matched with at first unfamiliar non-littermate WT and HET play partners. For each focus rat the genotype sequence of the play partners was counterbalanced so that they either played first with a WT play partner or with a HET play partner. Each focus rat played with both genotypes. Play partners were always from the same experimental housing condition as the focus rat. Play partners did not differ more than 10 g in weight.

#### 2.2.3. Test sequence

**FIGURE 1:**
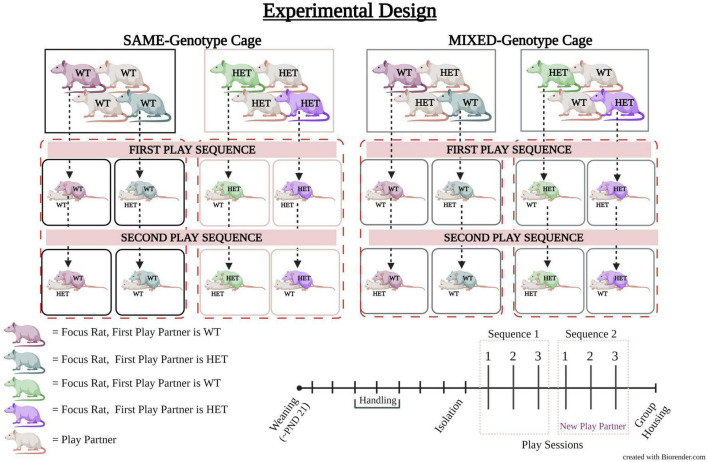
Experimental design of cage mate genotype and play partner genotype. The experimental design involved cages with female rats of the same genotype (SAME-genotype cage, shown on the **left** side). These cages consist of either all wildtype (WT) females (represented by black boxes) or all heterozygous (HET) females (represented by tan boxes). On the **right** side, there are the cages with a mix of genotypes (MIXED-genotype cages), with an equal representation of HET and WT females (represented by gray boxes). In the first play sequence, a focus rat is paired with either a WT or HET partner. In the second play sequence, the same focus rat interacts with a partner opposite to the one it played with in the first sequence. As indicated in the legend (bottom **left**) the color of each focus rat represents its first play partner. Play partners are shown in white. For example, the dark purple WT rat plays first with a WT play partner; the dark green WT rat plays first with a HET play partner; the purple HET rat plays first with a HET play partner; and the light green rat plays first with a WT play partner. Females from SAME-genotype cages always play with a partner from a SAME-genotype cage while females from MIXED-genotype cages always play with a partner from a MIXED-genotype cage. The timeline for the experiment is shown (bottom **right**) in which all animals were weaned around postnatal day (PND) 21 and put into manipulated housing conditions. All animals were handled and then isolated prior to the start of play testing. Play testing consisted of two play sequences each composed of three play sessions. A new play partner was given for the second play sequence. Following the last play session animals were placed back into group housing.

Testing consisted of two play sequences, each composed of three play sessions that took place over consecutive days (Figure 1). During the first play sequence, the focus rat played with a WT or HET partner for three consecutive days. On the fourth day, the play partners were changed to counterbalance across subjects and then the second play sequence commenced for the next three consecutive days. Consequently, during days four to six, each focus rat interacted with the converse partner compared to the preceding the 3 days, e.g., Day 1–3: WT-WT; Day 4–6: WT-HET (Figure 1). Importantly, the focus rats were presented with the same play partner within each 3-day sequence.

### 2.3. Rough-and-tumble play

Testing took place between PND 31 and 36. All rats were isolated the day prior to their first play session in a Makrolon type III cage (265 mm × 150 mm × 425 mm, plus high stainless-steel covers; Techniplast Deutschland GmbH) to enhance social motivation. In line with previous experiments (Kisko et al., 2018, 2020), play sessions were initialized by a 2-min anticipation phase, during which the focus rat habituated to the observation arena (35 cm × 35 cm, with Plexiglas walls; floor covered with 1 cm of fresh bedding). The anticipation phase was followed by a 5-min play phase, where focus and partner rats were allowed to socially interact. Before each play pair, the observation arena was cleaned thoroughly with an acetic acid solution (0.1%) and new bedding was added. Testing was conducted under red light conditions (∼28 lux). A digital camera (TK-1281 Color Video Camera, JVC, Yokohama, Japan) was used to record rats for behavioral analyses. Focus rats were marked with a non-toxic commercial marker, to enable the observer to distinguish the rats on the video.

Ultrasonic vocalizations recordings were taken during the play phase using UltraSoundGate Condenser CM16 Microphones connected to an UltraSoundGate 416H USB audio device (Avisoft Bioacoustics) placed 35 cm above the floor of the center of the observation arena. Briefly, acoustic data was recorded with a sampling rate of 250 000 Hz (recording range: 0–125 kHz; 16 bit) by Avisoft RECORDER USGH and transferred to Avisoft SASLab Pro for acoustical analysis. Start of behavioral recording was acoustically labeled by an audible beep signal from a stopwatch to synchronize audio and video recordings.

#### 2.3.1. Analysis of social play behaviors

The following behavioral measures were scored as play components or non-play components by a trained observer blind to the experimental conditions using the Observer XT (Noldus, Wageningen, Netherlands): Play components included pinning, wrestling, chasing, evasion, self-pinning, crawl-over, biting and nape attack. Non-play components consisted of sniffing, physical contact, self-grooming and grooming of the partner (see Table 1 for detailed descriptions). While evasion, self-pinning, crawl-over and biting were observed, they did not occur with sufficient frequency or specific playful meaning to contribute to the findings or to allow meaningful statistical analysis. Therefore, apart from being added to the total duration of play behaviors, they were not analyzed or discussed further.

**TABLE 1:** Definition of play and non-play behaviors as scored in the observation.

**Play behaviors**	**Pinning**	One rat lying with the dorsal surface on the floor with the other rat standing over it.
	**Wrestling**	A group of play-specific behaviors, including wrestling and boxing.
	**Chasing**	One rat moving in the direction of or pursuing the partner while the partner is moving away.
	**Nape Attack**	One rat actively nuzzles or pounces on the nape of the other.
	**Evasion**	The partner evades by running or jumping away.
	**Self-pinning**	One rat rotates to a supine position without preceding occurrence of an obvious attach by the other.
	**Crawl-over**	One rat crawls over the other rat.
	**Biting**	One rat bites and occasionally pulls the tail, ear, leg or fur of the other rat.
**Non-play behaviors**	**Sniffing**	One rat sniffs the other rats face, neck or anogenital area.
	**Grooming – self**	One rat licks paws, and cleans fur of themselves.
	**Grooming– other**	One partner actively grooms the other (licking, cleaning fur).
	**Physical contact**	One rat exhibits physical contact with the other without showing one of the other aforementioned behaviors.

While evasion, self-pinning, crawl-over, and biting was observed they did not occur with sufficient frequency and therefore, apart from being added to the total duration of play behaviors, are not analyzed or discussed.

#### 2.3.2. Analysis of USV

Using Avisoft SASLab Pro, 50-kHz and 22-kHz calls were manually counted for the entire 5-min play period in 20-s time bins by a trained observer. If two 50-kHz USV elements were at least 10 ms apart, two independent 50-kHz USV were counted. A frequency threshold of 32 kHz was defined to distinguish 50-kHz and 22-kHz USV. While atypical 50-kHz calls (Kisko et al., 2018) and 22-kHz calls were emitted, they did not occur with sufficient frequency and therefore were not analyzed or discussed further.

### 2.4. Statistical analysis

For comparing rough-and-tumble play behaviors and pro-social 50-kHz USV between genotype of the cage mates and genotype of the partner, analysis of variances (ANOVA) for repeated measurements were calculated with the between-subject factor genotype of the cage mates (H), genotype of the focus rat (G), genotype sequence of the play partner (i.e., WT or HET first) (SQ) and the within-subject factors genotype of the play partner (i.e., same or opposite) (P), play session (SE), rough-and-tumble play component (i.e., pinning, wrestling, chasing and nape attack) (C). *Post hoc* assessment of significant main effects was calculated using independent or paired *t*-tests, where appropriate. A significance threshold was set at *p* < 0.050.

## 3. Results

In the current study, we explored the interplay between acute and long-term effects of the genetic makeup related to the social environment and social peers on rough-and-tumble play and concomitant emission of 50-kHz USV during the critical developmental period in juvenile female rats. To this aim, we used a well-established genetic *Cacna1c* rat model and manipulated the genotype of the cage mates (SAME- vs. MIXED-genotype cages) and the genotype of the play partner (WT vs. HET play partner). Effects of *Cacna1c* haploinsufficiency were assessed in female constitutive heterozygous *Cacna1c*^+/–^ rats (HET: *N* = 40) and compared to wildtype *Cacna1c*^+/+^ littermate controls (WT: *N* = 40).

### 3.1. Rough-and-tumble play

**FIGURE 2:**
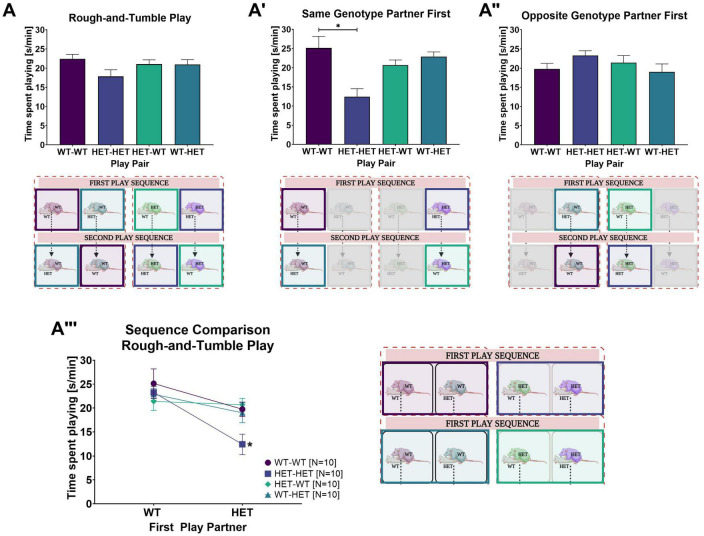
Rough-and-tumble play behavior and effects of the first play partner. The following data are pooled for genotype of the cage mate. Pair data included in the graphs are represented by the equivalent colored boxes surrounding the pairs of female rats in the representative design figure placed below or beside each graph (WT-WT = Dark Purple, HET-HET = Purple, HET-WT = Light Green, WT-HET = Dark Green, Greyed out boxes = not included). **(A)** The total averaged time spent playing for all six play sessions in pairs of female WT-WT (Dark purple bars, *N* = 20), HET-HET (Purple bars, *N* = 20), HET-WT (Light green bars, *N* = 20), and WT-HET (Dark green bars, *N* = 20). **(A’,A”)** The total averaged time spent playing across all three play sessions with a same- or opposite genotype play partner in pairs of female WT-WT (Dark purple bars, *N* = 10), HET-HET (Purple bars, *N* = 10), HET-WT (Light green bars, *N* = 10) and WT-HET (Dark green bars, *N* = 10). **(A”’)** The sequence comparison of time spent playing depending on genotype of the first play partner within female pairs of WT-WT (Dark purple circles, *N* = 10), HET-HET (Purple boxes, *N* = 10), HET-WT (Green diamonds, *N* = 10) and WT-HET (Dark green triangles, *N* = 10). Data are presented as mean + SEM. *p < 0.05.

**FIGURE 3:**
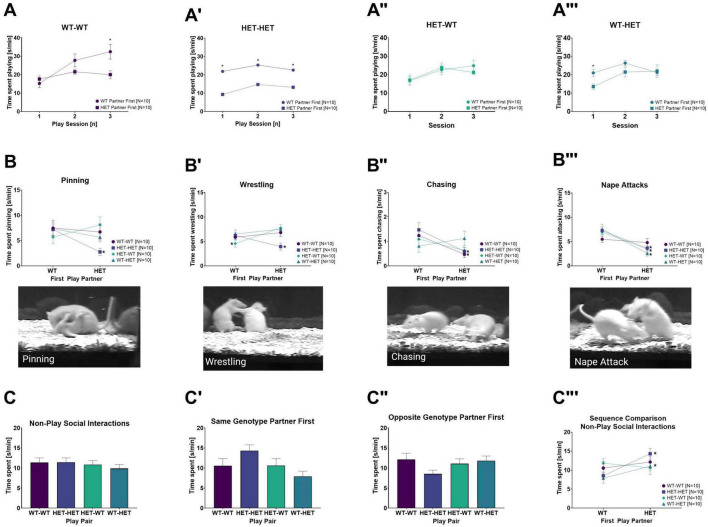
Rough-and-tumble play rough-and-tumble play components and non-play social interactions across play sessions. The following data are pooled for genotype of the cage mate. The total time spent playing across each individual play session depending on the genotype of the first play partner in **(A)** WT-WT, **(A’)** HET-HET, **(A”)** HET-WT and **(A”’)** WT-HET female pairs. The total time spent engaged and exemplary images for specific rough-and-tumble play components depending on genotype of the first play partner in female pairs of WT-WT (Dark purple circles), HET-HET (Purple boxes), HET-WT (Green diamonds) and WT-HET (Dark green triangles) for **(B)** pinning, **(B’)** wrestling, **(B”)** chasing and **(B”’)** nape attacks. **(C–C”’)** Non-play social interactions. **(C)** Total averaged time spent in non-play social interactions across all play session in pairs of female WT-WT (Dark purple bars, *N* = 20), HET-HET (Purple bars, *N* = 20), HET-WT (Light green bars, *N* = 20) and WT-HET (Dark green bars, *N* = 20). The total time spent averaged across play sessions in non-play social interactions with a **(C’)** same- or **(C”)** opposite genotype play partner in pairs of female WT-WT (Dark purple bars, *N* = 10), HET-HET (Purple bars, *N* = 10), HET-WT (Light green bars, *N* = 10), and WT-HET (Dark green bars, *N* = 10). **(C”’)** The sequence comparison of time spent in non-play social interactions depending on genotype of the first play partner in female pairs of WT-WT (Dark purple circles), HET-HET (Purple boxes), HET-WT (Green diamonds) and WT-HET (Dark green triangles). Data are presented as mean + SEM. *p < 0.05.

For juvenile WT and HET females, the amount of time spent engaged in rough-and-tumble play averaged across all play sessions was not dependent on the genotype of the cage mates, i.e., housing [PxH: *F*_(1,32)_ = 0.000, *p* = 0.990]. Because the genotype of the cage mate had no effect on play, the following results (Figures 2, 3) depict pooled housing conditions. However, the duration of rough-and-tumble play was dependent on the genotype of the play partner [PxG: *F*_(1,32)_ = 4.316, *p* = 0.046; Figure 2A and Table 2A]. Importantly, the effect of the play partner’s genotype on the time spent playing was strongly modulated by the sequence of the play partner (i.e., WT or HET play partner first).

**TABLE 2A:** *Post-hoc t*-test results for rough-and-tumble play behavior in pairs of juvenile female HET and WT rats.

Pair	Mean + SEM	*t*-test
WT-WT	22.4 + 1.8	t_18_ = 1.857, *p* = 0.071
HET-HET	17.8 + 1.7	
HET-WT	21.0 + 1.1	t_18_ = 0.060, *p* = 0.952
WT-HET	20.9 + 1.3	
*t*-test (HET-HET vs. HET-WT)	t_19_ = 1.716, *p* = 0.102	
*t*-test (WT-WT vs. WT-HET)	t_19_ = 0.903, *p* = 0.378	

Data are pooled for genotype of the cage mate and are shown as the mean seconds spent playing per minute + SEM. Section “3.1. Rough-and-Tumble Play.”

#### 3.1.1. Effects of the first play partner on rough-and-tumble play

When the first play partner was the same genotype, the time spent playing was much lower in same-genotype HET pairs than same-genotype WT pairs (WT-WT vs. HET-HET: Table 2B and Figure 2A’). In contrast, there was no significant difference between same-genotype HET pairs and same-genotype WT pairs when the first play partner was the opposite genotype (WT-WT vs. HET-HET: Table 2B and Figure 2A”), suggesting that playing first with a WT partner leads to an increase in the time spent playing in same-genotype HET pairs.

**TABLE 2B:** *Post-hoc t*-test results for rough-and-tumble play behavior in pairs of juvenile female HET and WT rats.

Pair First play partner	WT-WT	HET-HET	*t*-test (WT-WT vs. HET-HET)	HET-WT	WT-HET	*t*-test (HET-WT vs. WT-HET)
Same-genotype	25.1 + 3.1	12.4 + 2.1	t_18_ = 3.416, *p* = 0.003*	20.7 + 4.2	22.9 + 3.9	t_18_ = 1.202, *p* = 0.245
Opposite-genotype	19.7 + 1.5	23.3 + 1.3	t_18_ = 1.801, *p* = 0.088	21.4 + 1.9	19.0 + 2.1	t_18_ = 0.849, *p* = 0.407
*t*-test (same vs. opposite)	t_18_ = 1.581, *p* = 0.131	t_18_ = 4.407, *p* < 0.001*		t_18_ = 0.302, *p* = 0.766	t_18_ = 1.595, *p* = 0.128	

Data are pooled for genotype of the cage mate and are shown as the mean seconds spent playing per minute + SEM.

*Indicates a significant difference of *p* < 0.050. Section “3.1.1. Effects of the First Play Partner on Rough-and-Tumble Play.”

Indeed, the most important factor appears to be the sequence in which the focus rat plays with a same-or opposite-genotype partner [GxSQ: *F*_(1,32)_ = 10.694, *p* = 0.003; PxGxSQ: *F*_(1,32)_ = 6.682, *p* = 0.015]. A prominent rescue effect was found within same-genotype HET pairs. For same-genotype HET pairs the duration of play was higher when the first play partner was a WT compared to a HET (WT vs. HET: Table 2B and Figure 2A”’). In contrast, for same-genotype WT pairs, there was no difference in the time spent playing whether the first partner was a WT or a HET (WT vs. HET: Table 2B and Figure 2A”’). Within opposite-genotype pairs, no sequence effects were found (WT vs. HET: Table 2B and Figures 2A’–A”’). Of note, no main effects of genotype, housing, sequence, or other interactions were found (Supplementary Table 1A and Supplementary Figure 1C).

#### 3.1.2. Rough-and-tumble play across play sessions

A closer inspection of the time spent playing across individual play sessions between same- and opposite-genotype pairs showed an increase of the time spent playing across play sessions [SE: *F*_(2,64)_ = 26.584, *p* < 0.001]. For juvenile female WT and HET rats, the increase in play duration across sessions was dependent on the genotype and sequence of the play partners [PxSExG: *F*_(2,64)_ = 5.185, *p* = 0.008; PxSExSQ: *F*_(2,64)_ = 13.445, *p* < 0.001; PxSExGxSQ: *F*_(2,64)_ = 3.731, *p* = 0.029, a representation of unsorted and pooled sequence data across all six play sessions is shown in Supplementary Figure 1A].

##### 3.1.2.1. Effects of the first play partner on rough-and-tumble play across play sessions

In HET females playing with a same-genotype partner (HET-HET), the duration of playful interactions was nearly 50% higher in each individual play session (i.e., session 1, session 2, and session 3) when the first play partner was a WT compared to a HET (Table 2C and Figure 3A’). In HET females playing with an opposite-genotype partner (HET-WT), the time spent playing across sessions did not differ dependent on first play partner’s genotype (Table 2C and Figure 3A”).

**TABLE 2C:** *Post-hoc t*-test results for rough-and-tumble play behavior in pairs of juvenile female HET and WT rats.

Pair Session	WT-WT			HET-HET		
	**WT**	**HET**	***t*-test**	**WT**	**HET**	***t*-test**
1	15.2 + 2.3	17.6 + 1.7	t_18_ = 0.856, *p* = 0.404	21.9 + 1.6	9.3 + 1.1	t_18_ = 6.642, *p* < 0.001*
2	27.8 + 3.5	21.6 + 1.4	t_18_ = 1.653, *p* = 0.116	25.3 + 1.6	14.7 + 2.5	t_18_ = 3.556, *p* = 0.002*
3	32.4 + 4.0	20.0 + 2.2	t_18_ = 2.726, *p* = 0.014*	22.6 + 1.7	13.2 + 3.4	t_18_ = 2.472, *p* = 0.024*
** Pair** **Session**	**WT-HET**			**HET-WT**		
	**WT**	**HET**	***t*-test**	**WT**	**HET**	**t-test**
1	21.0 + 2.0	13.6 + 1.5	t_18_ = 2.971, *p* = 0.008*	16.7 + 2.3	17.1 + 2.7	t_18_ = 0.106, *p* = 0.916
2	26.3 + 1.4	21.5 + 2.7	t_18_ = 1.601, *p* = 0.127	22.6 + 2.6	23.8 + 2.5	t_18_ = 0.330, *p* = 0.746
3	21.3 + 1.6	22.0 + 3.4	t_18_ = 0.188, *p* = 0.853	24.8 + 3.1	21.2 + 1.4	t_18_ = 1.085, *p* = 0.292

Data are pooled for genotype of the cage mate and are shown as the mean seconds spent playing per minute + SEM.

*Indicates a significant difference of *p* < 0.050. Section “3.1.2.1. Effects of the First Play Partner on Rough-and-Tumble Play Across Sessions.”

In WT females playing with a same-genotype partner (WT-WT), more time was spent playing during the third play session when the first play partner was WT compared to a HET, but no effects were present in the first or second play sessions (Table 2C and Figure 3A). In WT females playing with an opposite genotype partner (WT-HET), less time was spent playing only in the first play session when the first play partner was a HET compared to a WT (Table 2C and Figure 3A”’).

#### 3.1.3. Rough-and-tumble play components

Detailed analysis of the specific play components showed that time spent pinning was most prevalent, followed by wrestling, nape attacks and chasing [C: *F*_(3,108)_ = 60.678, *p* < 0.001]. Genotype of the play partner (i.e., same- or opposite-genotype) did not affect the prevalence of the individual play components [P: *F*_(1,36)_ = 2.017, *p* = 0.164], yet there was an effect for the sequence of the play partner [PxSQ: *F*_(1,36)_ = 19.773, *p* < 0.001]. Moreover, an interaction between genotype of the play partner and genotype of the focus rat and sequence of play partner was also found [PxGxSQ: *F*_(1,36)_ = 5.375, *p* = 0.026]. Importantly, the duration for specific play components was dependent on the sequence of the play partner [CxGxSQ: *F*_(3,108)_ = 6.309, *p* < 0.001; PxCxSQ: *F*_(3,108)_ = 2.720, *p* = 0.048; PxCxGxSQ: *F*_(3,108)_ = 4.718, *p* = 0.004].

##### 3.1.3.1. Effects of the first play partner on rough-and-tumble play components

Like the duration of rough-and-tumble play, in HET females playing with a same-genotype partner (HET-HET), the duration of all playful components (i.e., pinning, wrestling, chasing, and nape attacks) was about 50% higher when the first play partner was a WT compared to a HET (Figures 3B–B”’ and Table 2D). When a HET female was playing with an opposite-genotype partner (HET-WT), less time was spent wrestling and more time was spent attacking the partner’s nape when the first play partner was a WT compared to a HET (Figures 3B–B”’ and Table 2D), while the duration of pinning and chasing was unaffected by the genotype of the first play partner.

**TABLE 2D:** *Post-hoc* t-test results for rough-and-tumble play behavior in pairs of juvenile female HET and WT rats.

Component	Pair	First play partner	Mean + SEM	*t*-test	Percent of Total Time Spent Playing^a^
**Pinning**	**WT-WT**	WT	7.5 + 1.5	t_18_ = 0.345, *p* = 0.734	28.3%
		HET	6.7 + 1.6		31.2%
	**HET-HET**	WT	7.2 + 1.1	t_18_ = 3.43, *p* = 0.003*	30.0%
		HET	2.8 + 0.7		22.7%
	**HET-WT**	WT	5.7 + 0.7	t_18_ = 1.189, *p* = 0.250	25.1%
		HET	8.1 + 1.5		36.9%
	**WT-HET**	WT	7.4 + 1.3	t_18_ = 1.036, *p* = 0.314	31.1%
		HET	5.7 + 0.9		29.8%
**Wrestling**	**WT-WT**	WT	5.9 + 0.5	t_18_ = 0.975, *p* = 0.343	25.6%
		HET	6.8 + 0.8		35.2%
	**HET-HET**	WT	6.2 + 0.6	t_18_ = 2.402, *p* = 0.027*	26.8%
		HET	4.0 + 0.7		32.4%
	**HET-WT**	WT	4.6 + 0.7	t_18_ = 3.194, *p* = 0.005*	20.9%
		HET	7.8 + 0.7		38.7%
	**WT-HET**	WT	6.5 + 0.8	t_18_ = 1.096, *p* = 0.288	28.7%
		HET	7.5 + 0.4		43.1%
**Chasing**	**WT-WT**	WT	1.2 + 0.2	t_18_ = 3.420, *p* = 0.003*	4.8%
		HET	0.5 + 0.1		2.2%
	**HET-HET**	WT	1.5 + 0.3	t_18_ = 2.628, *p* = 0.017*	6.2%
		HET	0.6 + 0.2		4.2%
	**HET-WT**	WT	1.1 + 0.3	t_18_ = 1.320, *p* = 0.203	4.7%
		HET	0.6 + 0.1		3.1%
	**WT-HET**	WT	8.1 + 0.3	t_18_ = 0.806, *p* = 0.431	3.2%
		HET	1.1 + 0.3		5.6%
**Nape attacks**	**WT-WT**	WT	5.5 + 0.6	t_18_ 0.649, *p* = 0.524	22.5%
		HET	4.8 + 0.4		26.0%
	**HET-HET**	WT	7.3 + 0.7	t_18_ = 2.699, *p* = 0.015*	32.2%
		HET	3.6 + 1.1		30.1%
	**HET-WT**	WT	6.9 + 0.6	t_18_ = 4.168, *p* < 0.001*	34.9%
		HET	3.5 + 0.6		17.5%
	**WT-HET**	WT	7.6 + 1.0	t_18_ = 4.320, *p* = 0.001*	34.2%
		HET	2.6 + 0.5		12.7%
	** Pair** **First play partner**	**WT-WT**	**HET-HET**	**HET-WT**	**WT-HET**
Total time spent playing across all play sessions (seconds/min)	**WT**	25.1 + 3.1	23.3 + 1.3	21.4 + 1.9	22.9 + 1.2
	**HET**	19.7 + 1.5	12.4 + 2.1	20.7 + 1.3	19.0 + 2.1

Data are pooled for genotype of the cage mate and are shown as the mean seconds spent playing per minute + SEM. *Indicates a significant difference of p < 0.050. Section “3.1.3.1. Effects of the First Play Partner on Rough-and-Tumble Play Components.”

^a^Proportion of the total time spent playing (across 5 min). The total time spent playing includes all play components listed in Table 1. Of note, the proportion of pinning, wrestling, chasing and nape attacks do not add up to 100% of total time spent playing. The remaining components listed in Table 1 account for this difference.

For WT females, only one play component appeared to be affected by the sequence of play partner. For same-genotype WT pairs (WT-WT), the duration of chasing was lower when the first play partner was a HET compared to a WT (Figures 3B–B”’ and Table 2D). When WT females played with an opposite-genotype partner (WT-HET), the time spent attacking the nape was reduced when the first play partner was a HET compared to a WT. The remaining play components were unaffected (Figures 3B–B”’ and Table 2D).

Between subjects, effects for rough-and-tumble play components between same- and opposite-genotype pairs showed an interaction effect of focus rat genotype and sequence of the play partner [GxSQ: *F*_(1,36)_ = 9.889, *p* = 0.003] but no main effect for the genotype of the focus rat or sequence of the play partner was observed [G: *F*_(1,36)_ = 1.672, *p* = 0.204; SQ: *F*_(1,36)_ = 0.329, *p* = 0.570].

### 3.2. Non-play social interactions

Within play sessions, the duration of non-play social interactions in WT and HET females depended on the play partner’s sequence [PxSQ: *F*_(1,32)_ = 7.909, *p* = 0.008]. However, with a closer look at the duration of non-play social behavior between pairs playing first with a same- or an opposite-genotype partner, no differences were found and are therefore not reported (Figures 3C–C”). As there were no significant differences in the duration of non-play social behaviors in same-and opposite-genotype pairs apart from being added to the total duration of non-play social interactions, individual components (Table 1) were not analyzed or discussed further. Additionally, the genotype of the cage mate had no effect on non-play social interactions and therefore the following results and representative figures (Figures 3C–C”’) are pooled for housing composition.

#### 3.2.1. Effects of the first play partner on non-play social interactions

When comparing the sequence of play partners within same- and opposite-genotype pairs for non-play social interactions, we observed that in HET females playing with a same-genotype partner (HET-HET) and WT females playing with an opposite-genotype partner (WT-HET), more time was spent in non-play social interactions when the first play partner was HET compared to WT (Table 2E and Figure 3C”’). No other effects of the first play partner were found in WT females playing with a same-genotype play partner (WT-WT) or in HET females playing with an opposite-genotype partner (HET-WT).

**TABLE 2E:** *Post-hoc t*-test results for rough-and-tumble play behavior in pairs of juvenile female HET and WT rats.

Pair First play partner	WT-WT	HET-HET	HET-WT	WT-HET
WT	11.1 + 1.8	8.5 + 0.9	11.1 + 1.7	7.9 + 1.3
HET	12.1 + 1.6	14.3 + 1.4	10.6 + 1.7	11.8 + 1.2
*t*-test	t_18_ = 0.671, *p* = 0.511	t_18_ = 3.343, *p* = 0.004*	t_18_ = 0.224, *p* = 0.825	t_18_ = 2.202, *p* = 0.041*

Data are pooled for genotype of the cage mate and are shown as the mean seconds spent interacting per minute + SEM.

*Indicates a significant difference of *p* < 0.050. Section “3.2.1. Effects of the First Play Partner on Non-Play Social Interactions.”

Between subjects, effects showed that the duration of non-play social interactions was dependent on the focus rats genotype and the play partner’s sequence [GxSQ: *F*_(1,32)_ = 4.610, *p* = 0.039]. However, the main effects for the genotype of the focus rat, the genotype of the cage mates and the genotype and sequence of the play partner did not affect non-play social interactions (Supplementary Table 1B).

### 3.3. Rough-and-tumble induced 50-kHz USV

**FIGURE 4:**
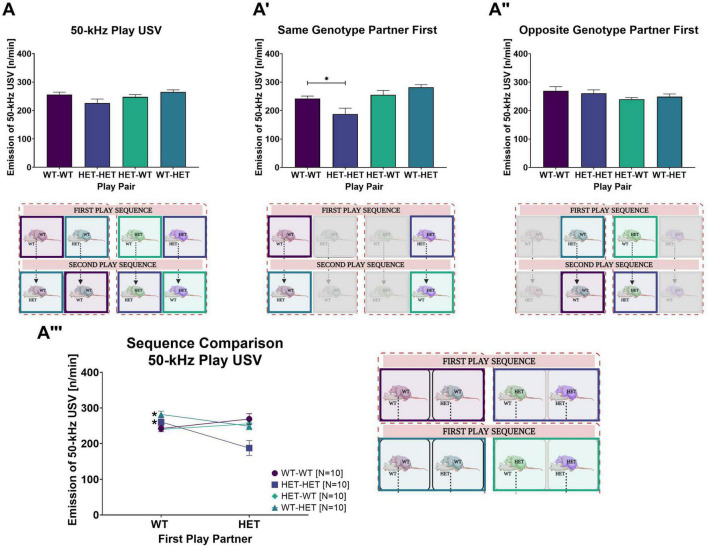
Rough-and-Tumble induced 50-kHz USV. The following data are pooled for genotype of the cage mate. Pair data included in the graphs are represented by the equivalent-colored boxes surrounding the pairs of female rats in the representative design figure placed below or beside each graph (WT-WT = Dark Purple, HET-HET = Purple, HET-WT = Light Green, WT-HET = Dark Green, Greyed out boxes = not included). **(A)** The total 50-kHz USV emissions averaged across all sessions in pairs of female WT-WT (Dark purple bars, *N* = 20), HET-HET (Purple bars, *N* = 20), HET-WT (Light green bars, *N* = 20) and WT-HET (Dark green bars, *N* = 20); **(A’,A”)** The number of 50-kHz USV emission (n/min) averaged across all sessions while playing with a same- or opposite genotype play partner in pairs of female WT-WT (Dark purple bars, *N* = 10), HET-HET (Purple bars, *N* = 10), HET-WT (Light green bars, *N* = 10) and WT-HET (Dark green bars, *N* = 10). **(A”’)** The sequence comparison of 50-kHz USV (n/min) emissions averaged across all sessions during social play depending on genotype of the first play partner in female pairs of WT-WT (Dark purple circles), HET-HET (Purple boxes), HET-WT (Green diamonds), and WT-HET (Dark green triangles). Data are presented as mean + SEM. *p < 0.05.

**FIGURE 5:**
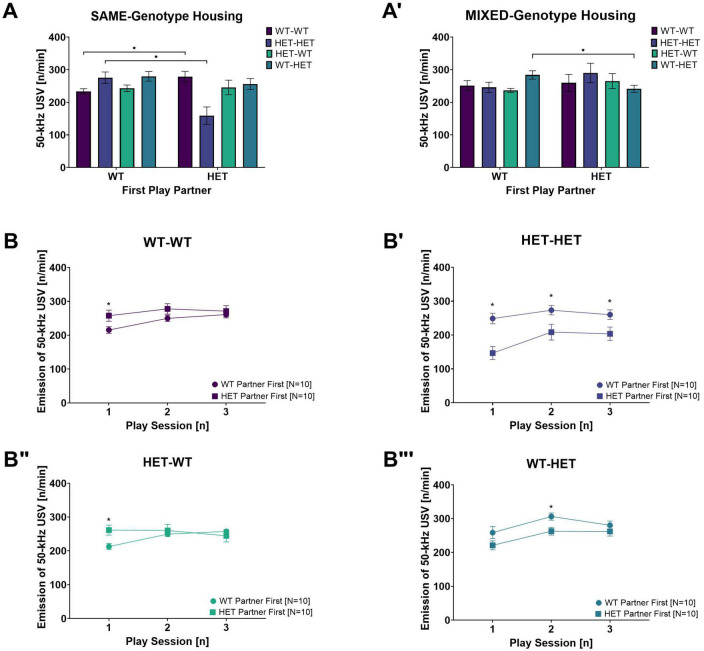
Rough-and-Tumble Induced 50-kHz USV across sessions and effects of the first play partner and housing. The total number of 50-kHz USV emissions per min averaged across all play sessions depending on the genotype of the first play partner in **(A)** SAME-genotype housing and **(A’)** MIXED-genotype housing for pairs of female WT-WT (Dark purple bars, *N* = 5), HET-HET (Purple bars, *N* = 5), HET-WT (Light green bars, *N* = 5) and WT-HET (Dark green bars, *N* = 5). **(B–B”’)** Data are pooled for genotype of the cage mate. The emission of 50-kHz USV across each individual play session depending on genotype of the first play partner (WT partner first = circles and HET partner first = squares) in female pairs of **(B)** WT-WT, **(B’)** HET-HET, **(B”)** HET-WT and **(B”’)** WT-HET; Data are presented as mean + SEM. *p < 0.05.

In slight contrast to rough-and-tumble play behavior, the total 50-kHz USV emission averaged across all play sessions in WT and HET females was not dependent on the genotype of the play partner [PxG: *F*_(1,31)_ = 0.717, *p* = 0.404, Figure 3A]. Instead, the emission of 50-kHz USV during rough-and-tumble play in same- and opposite-genotype pairs was dependent on the play partner’s sequence [PxSQ: *F*_(1,31)_ = 20.460, *p* < 0.001]. The genetic composition of the cage mates had no effect on the total averaged number of 50-kHz USV emissions across all play sessions. Accordingly, the *post-hoc t*-test results (Table 3A) and representative figures (Figures 4, 5B–B”’) for 50-kHz USV are pooled housing conditions.

**TABLE 3A:** *Post-hoc* t-test results for Rough-and-Tumble Play induced 50-kHz USV in pairs of juvenile female HET and WT rats.

Pair First play partner	WT-WT	HET-HET	*t*-test (WT-WT vs. HET-HET)	HET-WT	WT-HET	*t*-test (HET-WT vs. WT-HET)
Same-genotype	242.2 + 8.7	187.4 + 21.1	t_17_ = 2.504, *p* = 0.023	255.3 + 15.4	281.7 + 9.2	t_18_ = 1.478, *p* = 0.157
Opposite-genotype	269.0 + 9.6	260.8 + 12.10	t_18_ = 0.427, *p* = 0.674	239.9 + 5.6	248.7 + 9.8	t_18_ = 0.782, *p* = 0.444
*t*-test (same vs. opposite)	t_18_ = 1.545, *p* = 0.140	t_18_ = 3.103, *p* = 0.006*		t_18_ = 0.944, *p* = 0.358	t_18_ = 2.464, *p* = 0.024*	

Data are pooled for genotype of the cage mate. All data are shown as mean number of calls per min + SEM.

*Indicates a significant difference of *p* < 0.050. Section “3.3.1. Effects of the First Play Partner on 50-kHz USV.”

#### 3.3.1. Effects of the first play partner on 50-kHz USV

When the first play partner was the same genotype, the total 50-kHz USV averaged across all play sessions was lower in same-genotype HET pairs than same-genotype WT pairs (WT-WT vs. HET-HET: Table 3A and Figure 4A’). No effects of the first play partner on 50-kHz USV emissions was found in opposite-genotype pairs (WT-HET vs. HET-WT, Table 3A and Figure 4A”). Like play behavior, playing with a WT play partner appears to rescue the diminished 50-kHz USV emissions during rough-and-tumble play in same-genotype HET pairs.

Within each pair constellation, we saw that for same-genotype HET females (HET-HET), the decrease in 50-kHz USV was rescued when the first play partner was WT compared to the HET (WT vs. HET: Table 3A and Figure 4A”’). Notably, for pairs of HET females playing with a WT play partner (HET-WT), there was no differences in 50-kHz USV emissions, even if the first play partner was a HET compared to a WT (WT vs. HET: Table 3A and Figure 4A”’), strongly indicating that playing with a WT play partner is necessary for normalizing 50-kHz USV emissions during rough-and-tumble play in juvenile HET females. For WT females playing with a same-genotype partner (WT-WT), playing first with a WT or a HET does not affect the emission of 50-kHz USV (WT vs. HET: Table 3A and Figure 4A”’). However, in WT females playing with opposite-genotype play partners (WT-HET) higher 50-kHz USV emissions were seen when the first play partner was a WT compared to a HET (WT vs. HET: Table 3A and Figure 4A”’).

Between-subject comparisons showed a main genotype effect [G: *F*_(1,31)_ = 6.785, *p* = 0.014], with fewer 50-kHz calls associated with HET females compared to WT females. Furthermore, an interaction effect was observed between the cage mates’ genotype and the play partner’s sequence [HxSQ: *F*_(1,31)_ = 4.397, *p* = 0.044].

#### 3.3.2. Effects of the first play partner and genotype of the cage mate on 50-kHz USV

In SAME-genotype cages, the emission of 50-kHz calls in same-genotype pairs was higher when the first play partner was the opposite-genotype for both same-genotype WT and same-genotype HET pairs (WT-WT and HET-HET: Table 3B and Figure 5A). In contrast, no difference in 50-kHz USV emissions was observed in opposite-genotype pairs due to the sequence of play partner (WT-HET and HET-WT: Table 3B and Figure 5A). In MIXED-genotype cages, the opposite effect was found, same-genotype pairs showed no difference because of the sequence of the play partner (WT-WT and HET-HET: Table 3B and Figure 5A’), but opposite-genotype pairs showed higher 50-kHz USV emissions when their first play partner was of the same genotype (WT-HET and HET WT: Table 3B and Figure 5A’). The effect appears to be driven by opposite-genotype WT (WT-HET) pairs as the opposite-genotype HET (HET-WT) pairs, from MIXED-genotype cages, show no differences in 50-kHz USV emissions due to the first play partner (Table 3B and Figure 5A’).

**TABLE 3B:** *Post-hoc t*-test results for Rough-and-Tumble Play induced 50-kHz USV in pairs of juvenile female HET and WT rats.

SAME GENOTYPE CAGE MATES						
** Pair** **First play partner**	**WT-WT**	**HET-HET**	**t-test** **(WT-WT vs. HET-HET)**	**HET-WT**	**WT-HET**	***t*-test** **(WT-WT vs. HET-HET)**
Same-genotype	233.4 + 8.1	159.3 + 26.5	t_17_ = 3.751, *p* = 0.002*	245.6 + 22.3	279.7 + 14.7	t_18_ = 0.777, *p* = 0.447
Opposite-genotype	278.4 + 46.6	275.6 + 17.4	t_8_ = 2.050, *p* = 0.075	243.1 + 9.9	256.2 + 16.9	t_7_ = 3.305, *p* = 0.013*
*t*-test (same vs. opposite)	t_8_ = 2.437, *p* = 0.041*	t_7_ = 3.810, *p* = 0.007*		t_8_ = 0.102, *p* = 0.922	t_8_ = 1.054, *p* = 0.323	
**MIXED GENOTYPE CAGE MATES**						
** Pair** **First play partner**	**WT-WT**	**HET-HET**	**t-test** **(WT-WT vs. HET-HET)**	**HET-WT**	**WT-HET**	**t-test** **(WT-WT vs. HET-HET)**
Same-genotype	251.0 + 15.4	209.8 + 29.7	t_18_ = 0.986, *p* = 0.337	265.0 + 22.7	283.8 + 12.9	t_18_ = 2.537, *p* = 0.021*
Opposite-genotype	259.6 + 26.3	245.9 + 15.8	t_8_ = 0.493, *p* = 0.635	236.6 + 6.0	241.2 + 10.8	t_8_ = 0.407, *p* = 0.695
*t*-test (same vs. opposite)	t_8_ = 0.280, *p* = 0.787	t_8_ = 1.071, *p* = 0.316		t_8_ = 1.207, *p* = 0.262	t_8_ = 2.539, *p* = 0.035*	

All data are shown as mean number of calls per min + SEM.

*Indicates a significant difference of *p* < 0.050. Section “3.3.2. Effects of the First Play Partner and Genotype of the Cage Mate on 50-kHz USV.”

No main effect for the genotype of the cage mate or sequence of the play partner was found for same- and opposite-genotype pairs in the emission of 50-kHz USV (Supplementary Table 1C and Supplementary Figure 1D).

#### 3.3.3. 50-kHz USV across play sessions

Detailed investigation of the emission of 50-kHz USV across individual play sessions between same- and opposite-genotype pairs showed an increase of 50-kHz USV across all play sessions [SE: *F*_(1,62)_ = 36.819, *p* < 0.001].

##### 3.3.3.1. Effects of the cage mates genotype on 50-kHz USV across play sessions

The emission of 50-kHz USV across play sessions further showed an interaction effect for cage mate’s genotype, the focus rat’s genotype, and the play partner’s sequence [SExHxGxSQ: *F*_(1,62)_ = 3.671, *p* = 0.031]. In particular, the effect appears to be driven by higher emission rates of 50-kHz USV when the first play partner was the opposite-genotype in females with SAME-genotype cage mates. The effect is most prominent in the same-genotype HET pairs, which produced more rough-and-tumble 50-kHz calls when the first play partner was a WT (HET-HET: Table 3C). While in same-genotype WT pairs this effect was not significant (WT-WT: Table 3C). In pairs of juvenile females from MIXED-genotype cages, the emission of 50-kHz USV in same- and opposite-genotype pairs is roughly equivalent whether playing with the same-or opposite-genotype partner across most play sessions (Table 3C).

**TABLE 3C:** *Post-hoc t*-test results for Rough-and-Tumble Play induced 50-kHz USV in pairs of juvenile female HET and WT rats.

SAME GENOTYPE CAGE MATES						
** Pair** **Session**	**WT-WT**			**HET-HET**		
	**WT**	**HET**	**t-test**	**WT**	**HET**	**t-test**
1	42.5 + 2.1	53.4 + 4.7	t_8_ = 2.109, *p* = 0.068	54.5 + 3.7	26.7 + 2.9	t_8_ = 5.940, *p* < 0.001*
2	47.7 + 2.5	55.7 + 3.0	t_8_ = 2.050, *p* = 0.075	58.8 + 4.1	33.8 + 6.8	t_7_ = 3.305, *p* = 0.013*
3	49.8 + 1.3	57.9 + 2.9	t_8_ = 2.550, *p* = 0.034*	52.0 + 4.0	35.7 + 4.7	t_8_ = 2.642, *p* = 0.030*
** Pair** **Session**	**WT-HET**			**HET-WT**		
	**WT**	**HET**	**t-test**	**WT**	**HET**	**t-test**
1	47.5 + 5.5	46.4 + 2.9	t_8_ = 0.170, *p* = 0.869	42.4 + 2.7	54.3 + 3.6	t_8_ = 2.655, *p* = 0.029*
2	62.9 + 3.7	53.8 + 4.3	t_8_ = 1.603, *p* = 0.148	51.8 + 2.3	48.5 + 5.6	t_8_ = 0.549, *p* = 0.598
3	57.5 + 2.6	53.4 + 5.2	t_8_ = 0.689, *p* = 0.510	51.7 + 1.6	44.6 + 5.4	t_8_ = 1.267, *p* = 0.241
**MIXED GENOTYPE CAGE MATES**						
** Pair** **Session**	**WT-WT**			**HET-HET**		
	**WT**	**HET**	**t-test**	**WT**	**HET**	**t-test**
1	43.7 + 3.7	49.7 + 5.0	t_8_ = 0.962, *p* = 0.364	45.0 + 4.3	32.0 + 7.4	t_8_ = 1.515, *p* = 0.168
2	52.3 + 2.6	55.4 + 5.8	t_8_ = 0.493, *p* = 0.635	50.5 + 3.0	48.0 + 5.3	t_8_ = 0.407, *p* = 0.695
3	54.6 + 3.5	50.6 + 5.7	t_8_ = 0.600, *p* = 0.565	52.0 + 4.5	45.8 + 6.0	t_8_ = 0.829, *p* = 0.431
** Pair** **Session**	**WT-HET**			**HET-WT**		
	**WT**	**HET**	**t-test**	**WT**	**HET**	**t-test**
1	42.7 + 2.7	50.2 + 5.0	t_8_ = 1.330, *p* = 0.220	56.0 + 4.1	42.0 + 4.5	t_8_ = 2.292, *p* = 0.051
2	48.0 + 2.1	55.6 + 4.5	t_8_ = 1.529, *p* = 0.165	59.6 + 2.5	51.3 + 2.0	t_8_ = 2.532, *p* = 0.034*
3	51.3 + 1.6	53.2 + 4.3	t_8_ = 0.412, *p* = 0.691	54.7 + 4.4	51.4 + 2.5	t_8_ = 0.663, *p* = 0.526

All data are shown as mean number of calls per min + SEM.

*Indicates a significant difference of *p* < 0.050. Section “3.3.3.1. Effects of the First Play Partner and Genotype of the Cage Mate on 50-kHz USV Across Play Sessions.”

##### 3.3.3.2. Effects of the first play partner on 50-kHz USV across sessions

Like play behavior, the emission of 50-kHz USV across individual play sessions was dependent on the play partner’s genotype and sequence [PxSExSQ: *F*_(2,62)_ = 8.647, *p* < 0.001]. In same-genotype pairs (WT-WT and HET-HET), the emission of 50-kHz USV appears to be higher when the first play partner was the opposite-genotype compared to the same-genotype. Specifically, when the first play partner was a WT, the HET females playing with same-genotype partners showed higher 50-kHz USV emissions across three all play sessions (HET-HET: Table 3D and Figure 5B’). For same-genotype WT pairs a difference in emission of 50-kHz USV was evident only during the first play session when the first play partner was a HET compared to a WT (WT-WT: Table 3D and Figure 5B).

**TABLE 3D:** *Post-hoc t*-test results for Rough-and-Tumble Play induced 50-kHz USV in pairs of juvenile female HET and WT rats.

Pair Session	WT-WT			HET-HET		
	**WT**	**HET**	**t-test**	**WT**	**HET**	**t-test**
1	215.6 + 10.1	257.9 + 16.6	t_18_ = 2.185, *p* = 0.042*	248.8 + 15.6	146.8 + 19.2	t_18_ = 4.125, *p* < 0.001*
2	250.1 + 3.3	277.9 + 15.3	t_18_ = 1.552, *p* = 0.138	273.3 + 13.8	208.6 + 23.3	t_17_ = 2.448, *p* = 0.026*
3	261.1 + 9.7	271.2 + 16.2	t_18_ = 0.541, *p* = 0.105	260.1 + 14.3	203.8 + 19.8	t_18_ = 2.311, *p* = 0.033*
**Pair** **Session**	**WT-HET**			**HET-WT**		
	**WT**	**HET**	**t-test**	**WT**	**HET**	**t-test**
1	258.6 + 17.7	221.0 + 13.1	t_18_ = 1.707, *p* = 0.105	212.8 + 9.0	261.3 + 14.8	t_18_ = 2.800, *p* = 0.012*
2	250.2 + 10.8	262.8 + 11.5	t_18_ = 2.754, *p* = 0.013*	249.4 + 7.9	260.1 + 18.0	t_18_ = 0.545, *p* = 0.593
3	280.5 + 12.2	262.1 + 13.7	t_18_ = 1.000, *p* = 0.330	257.5 + 5.3	244.4 + 17.9	t_18_ = 0.700, *p* = 0.493

Data are pooled for genotype of the cage mate. All data are shown as mean number of calls per min + SEM.

*Indicates a significant difference of *p* < 0.050. Section “3.1.2.1. Effects of the First Play Partner on 50-kHz USV across Sessions.”

In contrast, the opposite-genotype pairs (WT-HET and HET-WT) showed higher 50-kHz USV emissions across each individual play session when the first play partner was the same-genotype. Within the pair constellations, the HET females playing with an opposite-genotype partner showed higher 50-kHz USV emissions only during the first play session, when the first play partner was a HET compared to a WT (HET-WT: Table 3D and Figure 5B”). In WT females playing with an opposite-genotype partner, there was a difference in 50-kHz USV emissions only during the second play session (WT-HET: Table 3D and Figure 5B”’).

## 4. Discussion

This study examined the interplay between acute and long-term effects of the genetic makeup related to the social environment and social peers on rough-and-tumble play and concomitant 50-kHz USV emission in juvenile female *Cacna1c* haploinsufficient rats and wildtype littermate controls. In contrast to what we expected based on our previous study (Kisko et al., 2020), we found that the cage mate’s genotype and the focus rat’s genotype had no main effects on play behavior and 50-kHz USV. What we found to be more important is the genotype and sequence of the play partner. Specifically, we found that same-genotype HET play pairs engage less in play behavior and emit fewer 50-kHz USV than same-genotype WT play pairs when first playing with a HET partner. Importantly, WT play partners can rescue social play and 50-kHz USV deficits in same-genotype HET pairs. The prominent rescue effect in HET rats by WT play partners indicates that early social environment and social peers significantly influence social behavior and communication development in juvenile female *Cacna1c* haploinsufficient rats.

### 4.1. Rough-and-tumble play

Play behavior is essential to development (Pellis and Pellis, 2009; Vanderschuren and Trezza, 2013; Vanderschuren et al., 2016). Juvenile rats are highly motivated to play and in the present study all pairs engaged in rough-and-tumble play, suggesting that motivation to play was present both in *Cacna1c* haploinsufficient females and wildtype littermate controls, irrespective of genetic makeup related to the social environment and social peers. However, we saw a reduced amount of time playing in same-genotype HET pairs, particularly if their first play partner was a HET. Interestingly, the time spent playing was rescued to levels equivalent to the same-genotype WT control pairs when a HET female played with a WT play partner regardless of sequence. Notably, this was evident not only in the average duration of play across all play sessions but also within each individual play session and relevant play components, i.e., pinning, wrestling, chasing and nape attacks. The WT females, therefore, can enhance the playful motivation of their HET play partners acutely and long-term, as the effect is persistent when the subsequent play partner is another HET.

The influence of the WT female rats on the HET contrasts our initial hypothesis that the hyper-playful HET females were responsible for increasing the playful motivation of their WT cage mates (Kisko et al., 2020). In rats, play partners and early social environment can affect the overall playful and social characteristics established during the critical period (Varlinskaya et al., 1999; Burgdorf et al., 2005; Himmler S. et al., 2014; Kisko et al., 2015a,b; Burke et al., 2017; Redecker et al., 2019; Stark and Pellis, 2020). Devocalization studies have shown that control rats living with devocalized cage mates spent less time playing than controls housed with other controls (Kisko et al., 2015b). Additionally, when rats are reared with adults or non-playful cage mates as juveniles, they show impairments in socio-cognitive functions (Bell et al., 2010; Baarendse et al., 2013; Himmler S. et al., 2014; Schneider et al., 2016; Pellis et al., 2017; Stark and Pellis, 2020) and are less socially competent when navigating adult social interactions (Kisko et al., 2015a; Stark and Pellis, 2020). In the current study, manipulating the post-weaning social environment had no significant main effects on the time spent playing for pairs of juvenile female rats. While it is important to acknowledge that the post-weaning social environment could have potentially influenced the individual play partners, it would be remiss not to consider its impact. During rough-and-tumble play in situations where it is possible, participants adapt their play behavior to their partner (Varlinskaya et al., 1999; Burgdorf et al., 2005; Himmler S. et al., 2014; Kisko et al., 2015b). In this manner, each partner benefits from the interaction. In adult Cacna1c haploinsufficient rats, we have found that the HET females will adapt their social behavior based on their partner’s genotype (Redecker et al., 2019). Our results indicate that the juvenile females adjust their behavior to suit their play partner’s genotype. However, it would be important to examine each playmate closely, particularly in opposite-genotype pairs. Additionally, the sequence in which the partners genotypes are presented plays a role in determining how playful the pair will be. For instance, HET females appear to be more playful when the first play partner was a WT, while WT females show more subtle effects when playing first with a HET play partner, i.e., spending less time playing only in certain play sessions and within select play components.

### 4.2. Non-play social behavior

In same-genotype HET and opposite-genotype WT pairs, there is an increase in non-play social behaviors when the genotype of the first play partner was a HET. In a study by Varlinskaya et al. (1999), the authors showed that in juvenile rats, the isolated, hyper-playful partner would adapt their behavior if their partner was reluctant to play and instead engage in more non-play-directed social behaviors (Varlinskaya et al., 1999). Therefore, it may be that the HET females are not as motivated to engage in rough-and-tumble play behaviors but rather prefer more non-play social interactions. If this were the case, we would also expect to see no difference in 50-kHz USV emissions, especially during periods of play, compared to non-play social interactions. At this point, however, we cannot say whether the HET individual drives the duration of non-play social interactions. We also cannot say during which behaviors 50-kHz USV are emitted, suggesting a more in-depth look and a detailed assessment of the individual play partners in combination with 50-kHz USV emissions.

### 4.3. 50-kHz rough-and-tumble USV

Like play behavior, the sequence of play partner affected the emission of 50-kHz USV in same-genotype HET pairs. When the first play partner was a WT, the same-genotype HET pairs emitted more 50-kHz USV and consequently showed no difference to the same-genotype WT control pairs. For the opposite-genotype pairs, the sequence of play partner had no effect on 50-kHz USV emissions and as such were comparable to same-genotype WT control pairs. Thus, for juvenile female rats the WT play partner appears to increase the emission of 50-kHz USV to control levels and appears to rescue deficits in 50-kHz USV emission in same-genotype HET pairs. Higher 50-kHz USV emissions in the same-genotype HET pairs after playing first with a WT or in opposite-genotype pairs are not altogether surprising, as they also spend more time playing. It is well-known that high rates of social play are associated with high rates of 50-kHz USV emissions in juvenile rats (Knutson et al., 1998; Burgdorf et al., 2005).

For same-genotype WT pairs, we again see mild effects of the first play partner. In fact, sequence effects are only evident within individual play sessions for 50-kHz USV emissions in WT females playing with a same- or opposite-genotype partner. Specifically, during the first play session same-genotype WT pairs emit more 50-kHz USV when the first play partner was a HET compared to a WT. In opposite-genotype WT pairs 50-kHz USV are reduced when the first play partner was a HET compared to WT, particularly within the second play session. The findings in WT pairs with the same and opposite-genotype partners are not too surprising because they can be attributed to the influence of the first play partner and the lasting effects it may have on play behavior. For instance, in half of the same-genotype WT pairs, the first play partner is another WT, while in the other half, they initially played with a partner of different genotype (HET). It is understandable, therefore, that they might be accustomed to playing with the HET partner in such cases. The reason for this is that the difference in behavior only occurs during the first play session. We can speculate that WT females may produce more 50-kHz USV initially to create a playful atmosphere possibly out of habitual tendency, even when they are paired with a new play partner. However, in subsequent sessions, they learn that this behavior is not necessary. Regarding the opposite-genotype WT pairs, the difference in 50-kHz USV emissions occurs only in the second play session. This might also be attributed to the influence of the previous play partner. In half of the pairs, the first play interaction involves a HET partner, whereas in the other half, they have already played with a WT partner for 3 days. As a result, they may still be highly motivated to play and, therefore, emit more 50-kHz USV to encourage their new HET play partner to engage in higher levels of playful behavior.

We know from devocalization studies that a vocal rat playing with a devocalized partner will increase the emission of 50-kHz USV to be comparable to pairs of two vocal rats leading to an increase in the playful mood and higher rates of play (Burke et al., 2017). Although, there is no way to say if the WT females are calling more than the HET females within the opposite-genotype pairs. However, it does not seem so unlikely based on the lower emission rates in same-genotype HET pairs.

Interestingly, while the genotype of cage mates did not affect play behavior, we found an interaction between the genotype of the cage mate and the sequence of the first play partner within 50-kHz USV emissions. In the females from SAME-genotype cages, the emission of 50-kHz USV was higher when the first play partner was the opposite genotype. Sequence effects in SAME-genotype cages are interesting as they reinforce the impression that WT females emit more 50-kHz USV when paired with HET play partners and that the HET females may adapt not only their behavior but also their social communication to complement the play partner leading to an increase in play and 50-kHz USV emissions in the subsequent play partners. In the MIXED-genotype housing condition, we see only an effect in the WT-HET pairs with higher 50-kHz USV when playing with a same-genotype partner first, which is likely driven by the increase in 50-kHz USV during the second play session. Thus, while not a primary effect, the genotype of the cage mate is not wholly unimportant as it provides the initial play experiences with and for both genotypes. For HET females, this is particularly important as it may rescue deficits that would be more prominent if housed with only other SAME-genotype HET cage mates.

There is strong evidence for social learning from conspecifics in various species, including rats (Carcea and Froemke, 2019). This includes social learning of food preferences (Galef Bennet, 2012) and observational fear learning (Kim et al., 2019). Moreover, we recently obtained evidence suggesting that juvenile rats may learn the appropriate use of USV within social contexts from their cage mates and play peers (Kisko et al., 2015a). By removing the ability to produce USV through devocalization, we found that devocalized juvenile rats reared with only other devocalized cage mates are not able to appropriately navigate ambiguous social interactions as adults, leading to increased aggression (Kisko et al., 2015a). In the present study, it is possible that the HET females may have learned from their WT cage mates, and vice versa.

Studies in mice have shown that social deficits in an ASD mouse model can be rescued by rearing them with a highly social mouse strain (Yang et al., 2011). From our study, similar effects might also occur in genetic rat models. The early social environment, through social peers and genotype of the cage mates, influences social communication in our *Cacna1c* juvenile HET and WT females. It provides further evidence to support findings in mice (Yang et al., 2015) that elements from the social environment, particularly the home cage, can interact with genotype to impact particular aspects of a disease model. Our findings reinforce the importance of the early social environment as a factor in phenotypic changes modulating genetic rat models of neuropsychiatric disorders.

In contrast to the current study, Yang et al. (2015) showed that mutant mice reared in mixed-genotype cages had fewer social interaction-induced USV than the wildtype controls, yet the mutant mice and wildtype controls reared in same-genotype cages did not differ in social interaction-induced USV emissions. The authors suggest that the genetic mutation is not necessarily causing the results but may be due to the mutant mouse’s subordinate status, which is eliminated in the same-genotype cage conditions (Yang et al., 2015). In *Cacna1c* adult females reared in mixed-genotype cages, we found reduced 50-kHz USV in same-genotype HET compared to same-genotype WT control and opposite-genotype pairs (Redecker et al., 2019). Although, in the social dominance tube test, the HET females were dominant over the WT females (Redecker et al., 2019), speaking somewhat against the results of our study being due to dominant-and subordinate roles within MIXED-genotype cages.

While we did not see any main effect of manipulating the cage mate’s genotype on the play behaviors, we did see an effect on 50-kHz USV emissions. Thus, the effects on behavior may be more subtle and become prominent when looking at the individual rats in each pair and how they respond to one another when initiating or responding to a playful attack. Social dominance in juvenile rats is typically measured through pinning behavior, with the more dominant rat pinning more (Panksepp et al., 1985). In the current study, the duration of play and play components are expressed as a value for the pair. Consequently, we cannot exclusively say at this moment what each specific partner is doing and if one is more dominant and thus pinning more than the other, but the analysis is on-going to establish the behavior of each rat. Furthermore, evidence from Pellis et al. (2022) shows that high and low-playing rats emit the same number of 50-kHz calls yet show differences in the call subtypes and associated behaviors. Thus, detailed investigations for the emission of 50-kHz USV during play would also provide more insight into the playful motivation and how they may use different communication signals depending on the partner’s genotype and the genotype of their cage mates.

### 4.4. Translational relevance

In humans, the *CACNA1C* gene has been linked through GWAS to an increased risk of ASD (Li et al., 2015), partly characterized by social and communication impairments. In the current study, the same-genotype HET pairs show social behavior and communication deficits reflected by reduced social play and 50-kHz USV emissions. However, there is a prominent rescue effect for the HET females by the WT play partners in behavior and 50-kHz USV. Our previous findings indicated that juvenile HET females have a hyper-playful repertoire and had male typical 50-kHz USV emission patterns (Kisko et al., 2020, 2021) yet still showed social communication impairments, particularly in aspects related to social incentive (Kisko et al., 2020). As adults, the HET females displayed further ASD-like social deficits and more repetitive behaviors than the WT controls (Redecker et al., 2019). Late emerging ASD-like phenotypes suggest first that rearing WT and HET juveniles together masks social deficits due to the rescue effect by WT cage mates and the innate drive for juveniles to engage in social play during this critical development period. Secondly, like humans (Baron-Cohen, 2002), ASD-like social impairments in *Cacna1c* haploinsufficient rats appears to manifest differently in males and females. It has been suggested that in humans autistic females may have fewer social impairments and show higher levels of social motivation than males (Head et al., 2014; Hiller et al., 2014), which is similar to what we have observed in the juvenile *Cacna1c* haploinsufficient rats.

Additionally, in female children a diagnosis of attention-deficit-hyperactivity disorder is often diagnosed years before a correct or accompanying diagnosis of ASD is recognized, due to the masking of social deficits (Rommelse et al., 2010). Thus, the alteration in Ca_v_1.2 protein levels may exert similar sex differences regarding ASD-like phenotypes in *Cacna1c* haploinsufficient juvenile rats, which may be supplemented by housing them with WT and HET cage mates in females. Furthermore, mixed-genotype housing in males may mask behavioral social deficits but lead to more noticeable deficits in social communication through USV, resulting in decreased social motivation and reduced 50-kHz USV emissions (Kisko et al., 2018). The housing arrangement with HET and WT cages mates, therefore, may have inadvertently rescued and potentially concealed social play and communication deficits in the HET females.

## 5. Conclusion

While the anticipated influence of the genetic composition of the social environment on rough-and-tumble play behavior was not observed, our findings demonstrate that MIXED-genotype housing provides an optimal context for HET females to engage in natural rough-and-tumble play interactions with WT partners. This valuable interaction effectively mitigates social deficits resulting from *Cacna1c* haploinsufficiency. Our study emphasizes the crucial role of genetic factors and social peers in shaping early social environments and their significant impact on the development of social behavior and communication in a rat model exhibiting ASD-like deficits.

## Data availability statement

The raw data supporting the conclusions of this article will be made available by the authors, without undue reservation.

## Ethics statement

The animal study was reviewed and approved by the Tierschutzbehörde, Regierungspräsidium Gießen, Germany.

## Author contributions

TK conceived the study with the help of MW. RK and TK performed the experiments. RB and TK analyzed the data. RB and TK wrote the manuscript with the help of MW, RS, and RK. RS and MW acquired funding. All authors contributed to the article and approved the submitted version.
