# Supplementary material for: Wildtype peers rescue social play and 50-kHz ultrasonic vocalization deficits in juvenile female Cacna1c heterozygous rats
Source: Front Behav Neurosci. 2023 Aug 3;17:1190272. doi: 10.3389/fnbeh.2023.1190272 (PMC10435996; doi:10.3389/fnbeh.2023.1190272)
Supplement: Supplementary file 1 [file Data_Sheet_1.docx]

Supplementary Material

# Supplementary Figures and Tables

## Supplementary Figures

**Supplementary Figure 1: Rough-and-Tumble Play and 50-kHz USV Across All Play Sessions and Effects of Housing on Rough-and-Tumble Play and 50-kHz USV**


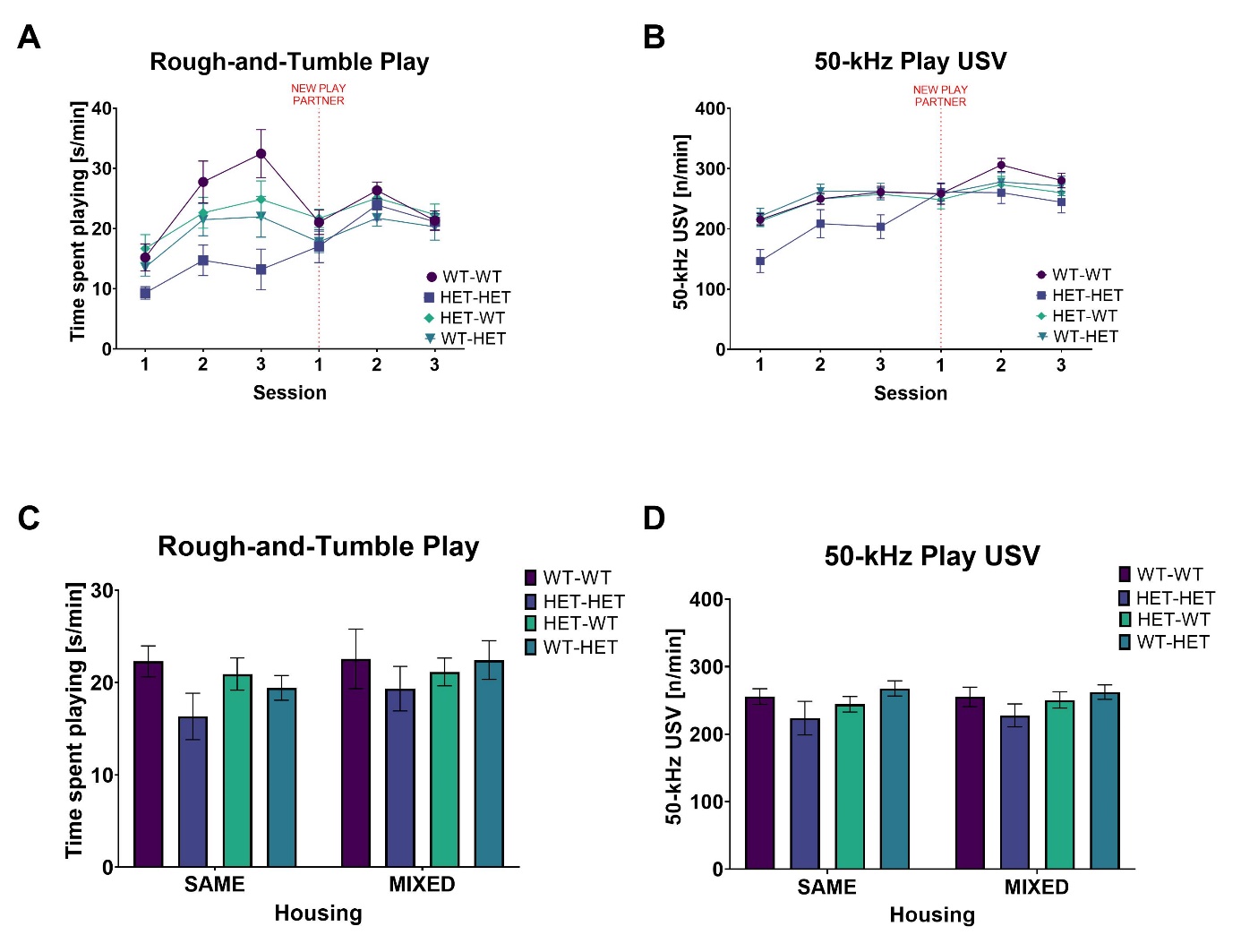


**Supplementary Figure 1: Rough-and-Tumble Play and 50-kHz Across all Play Sessions and Effects of Genotype of the Cage Mate on Rough-and-Tumble Play and 50-kHz USV**. **(A-B)** Data are pooled for genotype of the cage mate and for sequence of the play partner. **(**A) Total averaged per play session time spent playing across all six play sessions in pairs of female WT-WT (Dark purple circles, N = 10), HET-HET (Purple squares, N = 10), HET-WT (Light green diamonds, N = 10) and WT-HET (Dark green triangle, N, = 10). The red dotted line indicates the session where the new play partner was introduced. (B) Total averaged per play session 50-kHz USV emissions (n/min) across all six play sessions in pairs of female WT-WT (Dark purple circles, N = 10), HET-HET (Purple squares, N = 10), HET-WT (Light green diamonds, N = 10) and WT-HET (Dark green triangle, N = 10). The red dotted line marks the session where the new play partner was introduced, meaning the second play sequence began. (**C-D**) **Effects of** **Genotype of the Cage Mate on Rough-and-Tumble Play and 50-kHz USV**. (C) Total time spent playing averaged across all play sessions in pairs of female WT-WT (Dark purple bars, N = 10), HET-HET (Purple bars, N = 10), HET-WT (Light green bars, N = 10) and WT-HET (Dark green bars, N, = 10) depending on genotype of cage mates (SAME or MIXED); (D) Total 50-kHz USV emissions (n/min) averaged across all play sessions in pairs of female WT-WT (Dark purple bars, N = 10), HET-HET (Purple bars, N = 10), HET-WT (Light green bars, N = 10) and WT-HET (Dark green bars, N, = 10) depending on genotype of cage mates (SAME or MIXED). All data are shown as mean + SEM.

## Supplementary Tables

Supplementary Table 1: Between Subjects main effects from Analysis of Variance (ANOVA) for 1a: The duration of rough-and-tumble play across all play sessions. 1b: The duration of non-play social interactions across all play session and 1c: the number of 50-kHz USV emissions across all play sessions. Factors for between subject assessments included: genotype of the cage mates (H), genotype of the focus rat (G), genotype sequence of the play partner (i.e. WT or HET first) (SQ).

| **Supplementary Table 1a: 4.1.1 Main effects of Rough-and-Tumble Play Duration** | |  |
| --- | --- | --- |
| **Measure** | **Between Subject Effects** |  |
| Housing | H: F_1,32_ = 1.050, p = 0.313 |  |
| Sequence | SQ: F_1,32_ = 0.130, p = 0.720 |  |
| Genotype | G: F_1,32_ = 1.997, p = 0.167 |  |
| Housing x Genotype | HxG: F_1,32_ = 0.000, p = 0.995 |  |
| Housing x Sequence | HxSQ: F_1,32_ = 0.069, p = 0.795 |  |
| Housing x Genotype x Sequence | HxGxSQ: F_1,32_ = 0.010, p = 0.923 |  |
| **Supplementary Table 1b 4.2.1 Main effects of Non-Play Social Interactions** | |  |
| **Measure** | **Between Subject Effects** |  |
| Housing | H: F_1,32_ = 0.046, p = 0.832 |  |
| Sequence | SQ: F_1,32_ = 0.001, p = 0.972 |  |
| Genotype | G: F_1,32_ = 0.178, p = 0.676 |  |
| Housing x Genotype | HxG: F_1,32_ = 0.113, p = 0.739 |  |
| Housing x Sequence | HxSQ: F_1,32_ = 0.119, p = 0.732 |  |
| Housing x Genotype x Sequence | HxGxSQ: F_1,32_ = 1.856, p = 0.183 |  |
| **4.3.2 Supplementary Table 1c: Main effects of 50-kHz Rough-and-Tumble Induced USV** | | |
| **Measure** | **Between Subject Effects** | |
| Housing | H: F_1,31_ = 0.115, p = 0.736 | |
| Sequence | SQ: F_1,31_ = 2.061, p = 0.161 | |
| Housing x Genotype | HxG: F_1,31_ = 0.416, p = 0.524 | |
| Housing x Sequence | GxSQ: F_1,31_ = 3.067, p = 0.090 | |
| Housing x Genotype x Sequence | HxGxSQ: F_1,31_ = 0.497, p = 0.486 | |
